# Protocol for printing 3D neural tissues using the BIO X equipped with a pneumatic printhead

**DOI:** 10.1016/j.xpro.2022.101348

**Published:** 2022-04-22

**Authors:** Josie Chrenek, Rebecca Kirsch, Kali Scheck, Stephanie M. Willerth

**Affiliations:** 1 Department of Biomedical Engineering, University of Victoria, Victoria, BC V8W 2Y2, Canada; 2 Department of Mechanical Engineering, University of Victoria, Victoria, BC V8W 2Y2, Canada; 3 Division of Medical Sciences, University of Victoria, Victoria, BC V8W 2Y2, Canada; 4 School of Biomedical Engineering, University of British Columbia, Vancouver, BC V6T 1Z3, Canada

**Keywords:** Neuroscience, Stem Cells, Tissue Engineering, Biotechnology and bioengineering, Material sciences

## Abstract

3D bioprinting—a type of additive manufacturing—can create 3D tissue constructs resembling *in vivo* tissues. Here, we present a protocol for 3D printing neural tissues using Axolotl Biosciences’ fibrin-based bioink and the CELLINK BIO X bioprinter with a pneumatic printhead. This workflow can be applied to printing 3D tissue models using a variety of cell lines and any chemically crosslinked bioink. These 3D-printed tissue models can be used for applications such as drug screening and disease modeling *in vitro*.

## Highlights

• Protocol to 3D bioprint neural tissues with an extrusion-based bioink and the BIO X
• Prepares bioink and crosslinker that support many cell types including neural progenitors
• Protocol compatible with other cell types and chemically crosslinked bioinks

## Graphical abstract

**Figure:**
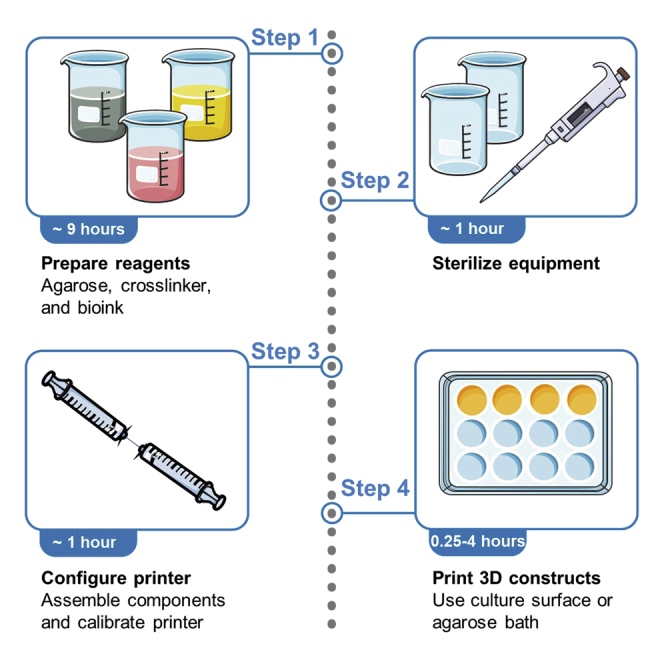

## Before you begin

This protocol describes the specific steps for preparing a cell-laden, chemically crosslinked bioink and an agarose support bath used for printing tissue constructs with the BIO X outfitted with a pneumatic printhead. The high viscosity, fibrin-based bioink and the crosslinker described in this protocol are the TissuePrint-HV Kit and TissuePrint Crosslink from Axolotl Biosciences. The bioink is adapted from one previously shown to support a variety of cell lines, including mesenchymal stem cells (MSCs) (Restan Perez et al., 2021) , neural progenitor stem cells (NPCs) (de la Vega et al., 2018; Abelseth et al., 2019; Sharma et al., 2020, 2021; de la Vega et al., 2021), glioblastoma (GBMs) (Smits et al., 2020), and human dermal fibroblasts (HDFs). The recommended passage number range is per the user’s individual cell line needs. Additionally, a protocol for preparation and printing of an Alg/Gel bioink is presented as an alternative bioink to use for printing tissue models.

Approximately 3–5 mL of crosslinker is required to crosslink 1 mL of bioink and these volumes can produce up to three cylindrical 10 × 10 mm prints at 25% infill. Shapes like domes, cylinders, and cubes are recommended based on the structural integrity and printability of the constructs. Approximately 1 million cells per mL of bioink is the suggested concentration for printing, equating to approximately 200,000–350,000 cells per construct. However, cell densities in the range of 500,000–5 million cells/mL are acceptable depending on the application.

This protocol can also be used to print constructs without cells by eliminating the cell incorporation steps. Such cell-free constructs might not require the sterilization steps depending on the application and can be eliminated accordingly. Additionally, the constructs can be printed directly onto a cell culture surface without the agarose support bath. The agarose preparation steps can be excluded in this situation. However, using the agarose support bath improves the structural integrity of the printed constructs. This protocol should be compatible with any bioink requiring chemical crosslinking after extrusion. A variety of synthetic and natural biomaterials exist that have been verified for extrusion bioprinting and should be compatible with this protocol. The bioink design should consider the mechanical, rheological, and crosslinking properties of the biomaterial components utilized (Benwood et al., 2021; Tarassoli et al., 2021; Yilmaz et al., 2021).

Although the following protocol presents several printing options, including procedures for an agarose support bath, different bioink types, and different printing surfaces, not all steps are required for every print. The agarose support bath is recommended for printing larger or more complex constructs to improve structural integrity. However, it is not required for highly viscous bioinks that can hold their shape well, such as Alginate/Gelatin (Alg/Gel) bioinks. Two bioinks are described in detail in this protocol: Axolotl Biosciences’ TissuePrint-HV bioink and an Alg/Gel bioink. However, these procedures can be applied to other chemically crosslinked bioinks. The protocol for printing with Alg/Gel is recommended for bioinks with a high viscosity that can maintain structural integrity without additional supports, while the protocol for the Axolotl Biosciences’ bioink using either pre-crosslinking or the support bath is recommended for lower viscosities. Finally, this protocol presents options for printing lower viscosity inks directly into a cell culture plate without a support bath, printing into an agarose support bath, and printing with higher viscosity inks like Alg/Gel that can adequately hold their shape without supports. Table 1 summarizes the steps described in this protocol where one option should be selected from each of the columns.

**Table 1:** Optional steps described in the following protocol, where one option should be selected from each column

Agarose support bath	Bioink type	Printing surface
Preparation of non-sterile agarose solution	Preparing the TissuePrint Crosslink solution, Preparing the TissuePrint-HV bioink with cells	Printing constructs directly onto a cell culture plate and pre-crosslinking
Preparation of sterile agarose solution	Preparing Alginate/Gelatin (Alg/Gel) bioink with cells	Printing constructs into an agarose support bath
None of the above		Printing with Alg/Gel

### Preparation of non-sterile agarose solution

**Timing: 8 h**

1. Place distilled water in a bottle with on a stir plate heated to 70°C with a stir rod. Add agarose while the bottle is being mixed at 350–400 rpm. The agarose cannot be microwaved.
  a. Typically, a mixture of 0.5% agarose is made for this application.
  b. Cover the bottle opening with a lid to prevent debris contamination and evaporation.
2. Mix the solution for 3–4 h until the agarose has completely dissolved, and the solution is no longer cloudy.
3. Once the agarose is dissolved, turn off the heat and increase the mixing speed to 900 rpm as the temperature of the agarose-water mixture drops to room temperature (approximately 20°C–22°C).
4. Keep the mixture on the hot plate for 3–4 h until it begins to thicken.
  a. When finished, the agarose mixture should have a smooth, translucent consistency (Figure 1A). The viscosity should be between that of water and syrup.

**Figure 1:**
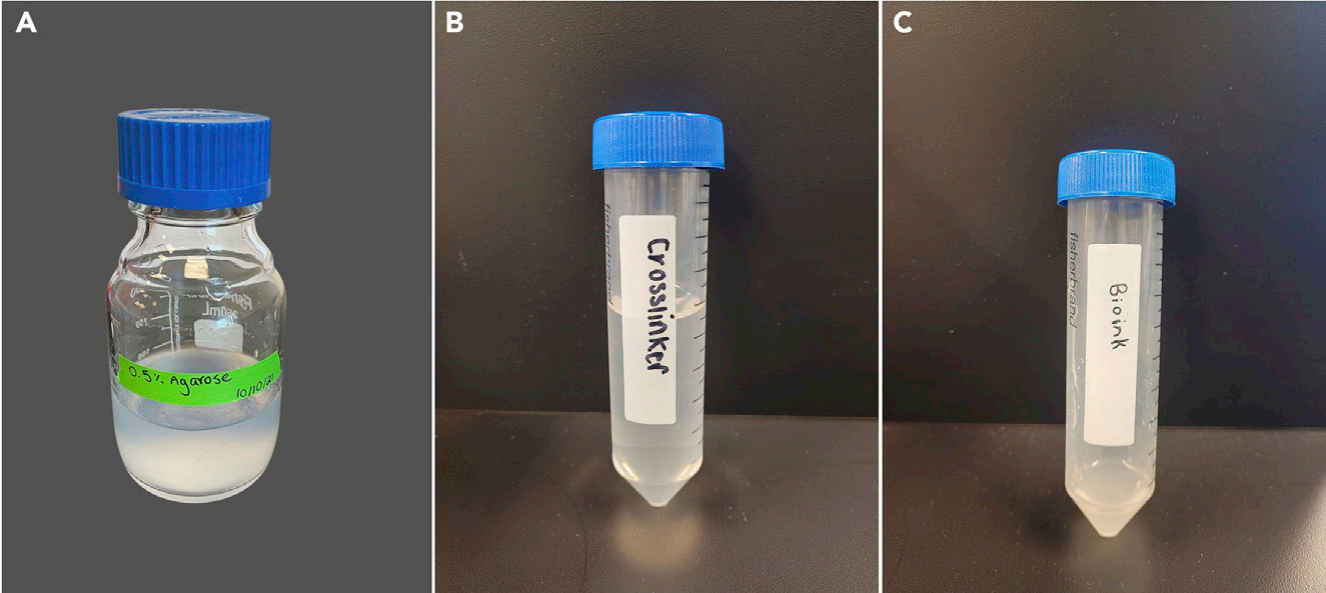
Representative images of the support bath materials along with the cross-linker and bioink (A) 0.5% agarose solution made in distilled water. Finished agarose has a smooth, translucent consistency. (B) Crosslinker should have a clear, colorless, and water-like consistency. (C) Bioink should have an opaque, slightly viscous consistency and contain no air bubbles.
5. Non-sterile agarose can be stored at 4°C for up to one week.

### Preparation of sterile agarose solution

**Timing: 8 h**

6. Place distilled water in an autoclavable bottle with a stir rod and add agarose.
7. Autoclave the solution at 121°C for 30 min.
8. While temperature is still above 80°C, remove from autoclave and immediately place on a hot plate set at 900 rpm.
9. Mix the solution for 3–4 h until it begins to thicken.
10. Sterile agarose can be stored at 4°C for up to one week.

### Preparing the TissuePrint Crosslink solution

**Timing: 30 min**

11. Thaw Component A and Component B of the TissuePrint Crosslink for 3–4 h at 4°C prior to mixing crosslinker.

***Note:*** The TissuePrint Crosslink components come pre-sterilized.

12. Using a pipette, slowly add Component B to Component A.

***Note:*** Each TissuePrint Crosslink kit is shipped in pre-measured volumes to make 20 mL of crosslinker. 20 mL of crosslinker is recommended for every 5 mL of bioink, which can produce up to three cylindrical 10 × 10 mm prints at 25% infill.

13. Pipette up and down to mix the solution.
14. If using crosslinker solution immediately after preparation, place it on ice until ready to use. Alternatively, store the crosslinker solution at 4°C for up to one week. The mixed crosslinker can be stored at a lower temperature, so long as it does not cause the crosslinker to freeze.

***Note:*** The crosslinker should have a clear, colorless, and water-like consistency when mixed (Figure 1B).

### Preparing the TissuePrint-HV bioink with cells

**Timing: 1.5 h**

15. Thaw TissuePrint-HV components prior to mixing bioink.
  a. Component 1 and Component 3 can be thawed at 4°C one day before using.
  b. Component 2 can be thawed at room temperature (approximately 20°C–22°C) immediately before using.

***Note:*** The TissuePrint-HV components come pre-sterilized.

16. Using a 200 μL pipette, add Component 2 to Component 1
17. Pipette up and down to mix using a wide bore pipette.
18. Obtain cells from frozen vials or culture dishes.
  a. Determine the number of vials or confluent culture dishes required for printing. 1 million cells per mL of bioink, or approximately 200,000–350,000 cells per construct, is recommended.
  b. Add cell media to thawed or detached cells to reach a total volume of 10 mL. Gently pipette up and down to obtain a cell suspension.
  c. Centrifuge cells at 300 g for 5 min or at the speed and time designated by cell culturing protocols.
  d. Remove the supernatant by aspiration.
19. Pipette the Component 2 directly onto the cell pellet and gently pipette up and down to resuspend cells.
20. Immediately before printing, add Component 3 to Component 1.
  a. Pipette up and down to create a homogenous solution.

***Note:*** Pipette the bioink slowly to avoid creating air bubbles. The bioink should have an opaque, slightly viscous consistency (Figure 1C).

**CRITICAL:** The bioink can be used for printing up to 1 h after combining the components. The components should not be combined a significant time before printing as they will cause flocculation, causing fibrin discs to form. Similarly, other bioinks can become clumped and difficult to extrude if components are combined too far in advance of printing. It is recommended to prepare any bioink as close to printing time as possible and to monitor the consistency of the ink for clumps before printing.

### Preparing alginate/gelatin (Alg/gel) bioink with cells

**Timing: 1 h**

21. Place distilled water in a beaker with a stir rod on a hot plate at 300 rpm.
22. Add in desired amount of alginate and gelatin.
  a. A mixture of 2% alginate/2% gelatin (w/v) is typically produced.
  b. Add gelatin to mixture first.
  c. Once gelatin is fully dissolved, add alginate.
  d. Cover the beaker to prevent debris and evaporation.
23. Heat mixture up to 135°C while mixing constantly until solution is fully incorporated, increasing the speed as needed.
  a. The solution should be as viscous as toothpaste and should be an amber color.
  b. Coloring, such as phenol red, can be added to enhance visibility while printing.
24. Transfer the solution to a conical and centrifuge at 300 g for 5 min to remove any bubbles from the solution to the surface.
25. Scrape the bubbles from the top off the solution.
26. Finished Alg/Gel can be stored at 4°C for up to 2 weeks. The bioink can be stored at a lower temperature, so long as it does not cause the bioink to freeze.

### Printing preparation

**Timing: 1 h**

27. Autoclave any lab equipment before printing.
  a. When printing in an agarose support bath, we recommend using two conical racks, two spatulas, a wash bottle, a 1 mL pipette, and two waste beakers (Figure 2A).

**Figure 2:**
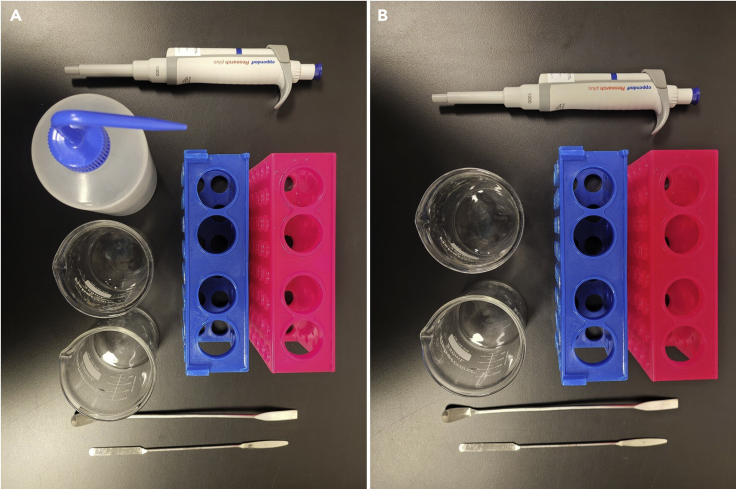
Laboratory equipment necessary for bioprinting tissue models (A) Recommended laboratory equipment for printing into an agarose support bath. (B) Recommended laboratory equipment for printing directly into a cell culture plate and pre-crosslinking.
  b. When printing directly onto a cell culture plate and pre-crosslinking, we recommend using two conical racks, two spatulas, a 1 mL pipette, and two waste beakers (Figure 2B).
28. Place BIO X printer in a sterile biosafety cabinet (BSC) and plug in the power cord.
29. Decontaminate the BIO X and BSC.
  a. We recommend using MycoClean Mycoplasma Prevention Spray, Conflikt™, and 70% ethanol.
30. Turn on the BIO X printer using the power switch located on the back panel (on the right-hand side when viewing from the front of the printer).
31. Perform UV sterilization of the BIO X chamber.
  a. Open the “Utilities” menu (the gear icon in the top right corner of the BIO X touchscreen).
  b. Select “Clean chamber”.
    i. Turn on “Clean chamber fan”.
  c. Click “Start” to begin the sterilization process.
32. UV the entire BSC using either it’s built in UV light or an external light source.

**CRITICAL:** Ensure the protective door of the BIO X is closed during the sterilization process to avoid UV damage to user’s eyes. When sterilizing the entire BSC, ensure UV protective safety glasses are worn.

## Key resources table

**Table:** 

REAGENT or RESOURCE	SOURCE	IDENTIFIER
**Chemicals, peptides, and recombinant proteins**		

TissuePrint Crosslinker	Axolotl Biosciences	https://www.axolotlbiosciences.com/product-page/tissueprint-crosslinker
TissuePrint-HV Kit	Axolotl Biosciences	https://www.axolotlbiosciences.com/product-page/neurobio-ink
Alginic acid sodium salt	Sigma-Aldrich	Cat#180947-100G
Gelatin from porcine skin	Sigma-Aldrich	Cat#G6144-100G
Agarose	Froggabio	Cat#A87-500G, CAS: 9012-36-6
Tris hydrochloric acid	VWR	Cat#VWRB85827-1KG
Tris(hydroxymethyl)aminomethane	Sigma-Aldrich	Cat#252859
Sodium chloride	Fisher Chemical	Cat#5674401KG
Potassium chloride	Caledon Laboratories	Cat#7447-40-7
MycoClean Mycoplasma Prevention Spray	Bulldog Bio	Cat#103507-506
Decon™ Conflikt™ Detergent Disinfectant	Fisher Scientific	Cat#04-355-34

**Experimental models: Cell lines**		

1-DL-01 (male) hiPSCs	WiCell	N/A
Human Adipose-derived MSCs	ScienCell™	Cat#7510
U-87 MG GBMs	ATCC	ATCC HTB-14™

**Other**		

BIO X	CELLINK	Cat#D16110020717
Empty cartridges with end and tip caps, 3 mL	CELLINK	Cat#CSC010300102
Sterile standard conical bioprinting nozzles, 22G	CELLINK	Cat#NZ4220005001
Female/female luer lock adapter	CELLINK	Cat#OH000000010
5cc Luer Lock syringe w/o Needle	Terumo Medical Corporation	Cat#SS-05L

## Materials and equipment

**Table:** 2% alginate/2% gelatin (w/v) (Alg/Gel)

Reagent	Final concentration	Amount
Gelatin from porcine skin	2%	200 mg
Alginic acid sodium salt	2%	200 mg
dH_2_O	n/a	10 mL
**Total**	**n/a**	**10 mL**

Prepared Alg/Gel solution can be stored at 4°C for up to 2 weeks. The solution can also be stored at lower temperatures, so long as it does not cause the solution to freeze.

**Table:** Tris-buffered saline (TBS)

Reagent	Final concentration	Amount
Tris hydrochloric acid	0.436%	17.44 g
Tris(hydroxymethyl)aminomethane	0.064%	2.56 g
Sodium chloride	0.8%	32 g
Potassium chloride	0.02%	0.8 g
dH_2_O	n/a	4 L
**Total**	**n/a**	**4 L**

Prepared TBS can be stored at room temperature (approximately 20°C–22°C) for up to 1 week or 4°C for up to 2 weeks.

## Step-by-step method details

### Preparing the BIO X with the bioink

**Timing: 15 min**

This portion of the protocol includes instructions to load the BIO X printhead and complete the printer setup before printing. Proper preparation of the BIO X, including loading the printheads and using correct printer parameters, will result in production of 3D biological constructs with the highest viability and quality.

***Note:***Methods video S1 demonstrates the following steps for setting up the BIO X printer with the bioink.

**Figure 3:**
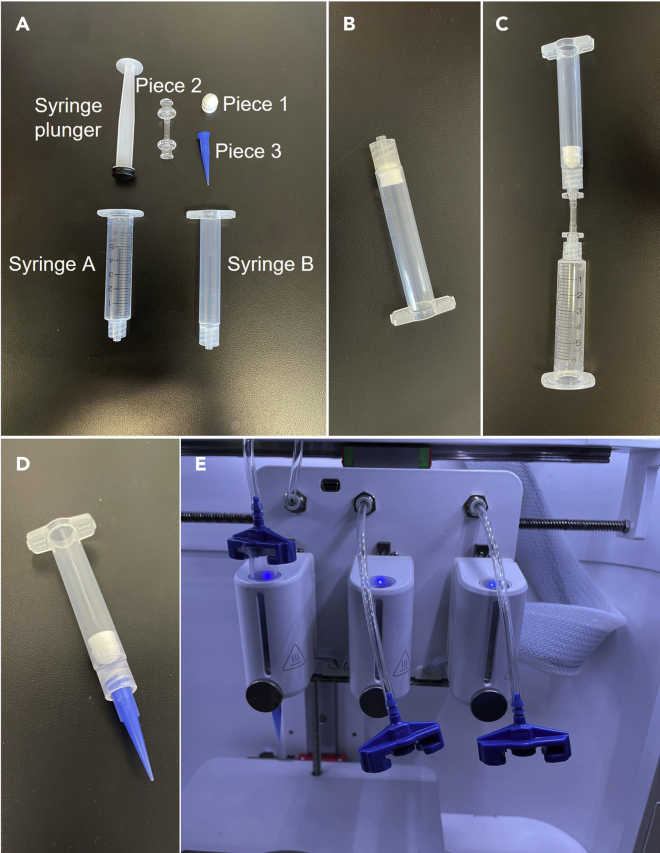
BIO X printhead and syringe setup (A) Pieces required for printhead syringe setup. (B) Syringe B with piece 1 (step 1a). (C) Setup required to fill syringe (step 1b). (D) Final set-up of syringe B (step 3). (E) Final set-up of syringe B and printhead (step 4).

1. Set up the printhead (Figure 3).
  a. Place ‘piece 1’ (Figure 3A) into syringe B and use a thin spatula to push it to the bottom of the syringe (Figure 3B).
    i. Piece 1 should be level inside the syringe and air should not be able to escape between the syringe and piece 1
      ***Note:*** Syringe B and piece 1 are the empty cartridges with end and tip caps in the key resources table.
  b. Attach syringe A to syringe B via piece 2 (Figure 3C).
  c. Pour the bioink into syringe A.
    i. Avoid creating or adding bubbles into the syringe. Problem 1: Air bubble formation.
    ii. A small scraper can be used to remove the remaining bioink from the conical.

2. Use the syringe plunger to push the bioink through piece 2 and into syringe B.
3. Remove piece 2 and attach piece 3 to syringe B (Figure 3D).

***Note:*** When printing into agarose, attach a blunt tip needle to syringe 2 instead of piece 3.

4. Insert syringe B into the printhead, referred to as “Tool 1” on the BIO X (Figure 3E).
  a. Attach syringe B to the pressure hose via the pressure connector above the printhead mount. Twist the pressure connecter to attach to syringe B.
  b. Insert syringe B into the printhead by pushing down until the syringe is fully inserted.

Methods video S1. Demonstration of BIO X printer setup with bioink, related to preparing the BIO X with the bioink steps 1–4

### Choosing a design and printing parameters

**Timing: 15 min**

5. Select “Bioprint” on the BIO X touchscreen.
6. Choose the desired model from the “Model” tab.
  a. Shapes such as domes, cubes, and cylinders are recommended for structural integrity and printability.
  b. Up to three 10 × 10 mm cylinder prints at 25% infill can be printed using 1 mL of bioink and 3–5 mL of crosslinker.

***Optional:*** New designs can be uploaded to the BIO X using the USB port on the front panel of the printer.

7. Select the “Surface” tab and adjust the surface properties.
  a. Choose the desired printing surface (e.g., Petri dish, well plate), size, and vendor (if applicable).
    i. If using a well plate, select the well plate icon to set the printing pattern and desired wells.
    ii. If printing into an agarose support bath, the 6 well culture plate is recommended.
8. Select the “Printer” tab and adjust the printing properties.
  a. Select the printhead being used (e.g., Tool 1 if using the printhead on the far left).
  b. Set “Pneumatic 3 mL” as the tool type.
  c. Set Bioink profile to “CELLINK START”.
  d. Ensure Photo-crosslinking is set to “Off”.
  e. Choose a desired pressure and printing speed.
    i. When printing into an agarose support bath, a pressure of 10 kPa and a speed of 15 mm/s is suggested for the TissuePrint-HV kit.
    ii. When printing directly into a cell culture plate and pre-crosslinking, a pressure of 5 kPa and a speed of 10 mm/s is suggested when printing with TissuePrint.
9. Select the “Layers” tab and adjust layer properties.
  a. Choose a desired layer profile.
    i. “Grid Lattice” is recommended.
  b. Select a desired infill pattern.
  c. Set desired infill density.
10. Select the “Print” tab and confirm printing settings.
  a. Press “Save” to save printing parameters, if desired.
  b. Press “Print” to begin printing when satisfied with printing settings.

***Note:*** For images of the BIO X setup, refer to the BIO X user manual (BIO X user manual, 2019).

### Calibrating the BIO X

**Timing: 15 min**

11. Select “Calibrate” at the bottom of the screen.
  a. Choose “Manual Calibration” followed by the “Start” button.
12. Adjust the parameters until the needle height is the desired distance from the printing surface and press “OK”.
  a. When printing into an agarose support bath, it is recommended to calibrate the needle tip to approximately 1 mm from the bottom of the well.
  b. When printing directly into a cell culture plate and pre-crosslinking, it is recommended to calibrate the nozzle tip to approximately 0.1 mm from the bottom of the well.
13. Test pressure before beginning print to check extrusion.
  a. Select “Pressure” from the menu.
  b. Select pressure tab.
  c. Hold down the “Test Flow” button to test extrusion.
    i. If needed, adjust the pressure until the bioink extrudes at a rate of approximately 1 drop/second.

***Note:*** Clean off the nozzle after pressure and speed testing by gently wiping with a Kimwipe™ sprayed with ethanol before printing to remove any excess biomaterial.

14. Press “Print”.

***Note:*** Refer to the BIO X User Manual for further information on printing parameters and calibration protocols manual (BIO X user manual, 2019).

### Printing constructs directly onto a cell culture plate and pre-crosslinking

**Timing: 20 minto4 h**

This step includes the instructions on how to print 3D tissue constructs directly onto cell culture plates. This method is suitable for more viscous materials that can hold their shape. Table 2 presents the recommended printing parameters for this method.

15. Begin the print by pressing “Print”.
  a. The printhead tip should be just above (∼0.1 mm) the cell culture plate/Petri dish.
  b. Adjust the speed and pressure as needed to obtain a steady and even print. Problem 2: Lack of or inconsistent material extrusion, problem 3: Nozzle tip becomes clogged.

***Optional:*** If the printed construct does not hold its shape during printing, coat the well with crosslinker before printing. Troubleshooting 4: Printed construct does not hold its shape during printing.

16. When the print is finished, add crosslinker to the cell culture well using a pipette. Problem 5: Nozzle tip leaks between prints.
  a. Pipette crosslinker using a micropipette onto the edges of the well. Do not add crosslinker directly onto the 3D printed construct as this will distort the construct shape.
  b. Add enough crosslinker to submerge the construct.
17. Let the construct sit in the crosslinker for 2–3 min or until the printed construct has sufficiently crosslinked.

***Note:*** The printed construct is fully crosslinked when the material has gelled and holds its shape.

***Note:*** The crosslinking time may vary depending on the bioink formulation being used.

18. Remove the crosslinker using a pipette.
19. Transfer the construct to a new (pre-coated, if applicable) cell culture plate and fill the wells with cell culture media. If printing without cells, tris-buffered saline (TBS) can be used. Preparation of TBS has been previously outlined by our lab (Abelseth et al., 2019).

***Optional:*** TBS or media can be added to the wells of the original cell culture plate instead of transferring the construct to a new plate if a pre-coated plate is not required .

**Table 2:** Recommended printing parameters for printing directly onto a cell culture plate

Printing parameter	Value
Calibration Height	0.1 mm
Print Speed	15 mm/s
Pressure	11 kPa
Crosslinking Time	2–3 min

### Printing constructs into an agarose support bath

**Timing: 20 minto4 h**

This step includes the instructions on how to print 3D tissue constructs into an agarose support bath. This method is better suited for materials and structures that do not hold their shape well directly after printing. Table 3 presents the recommended printing parameters for this method.

**Figure 4:**
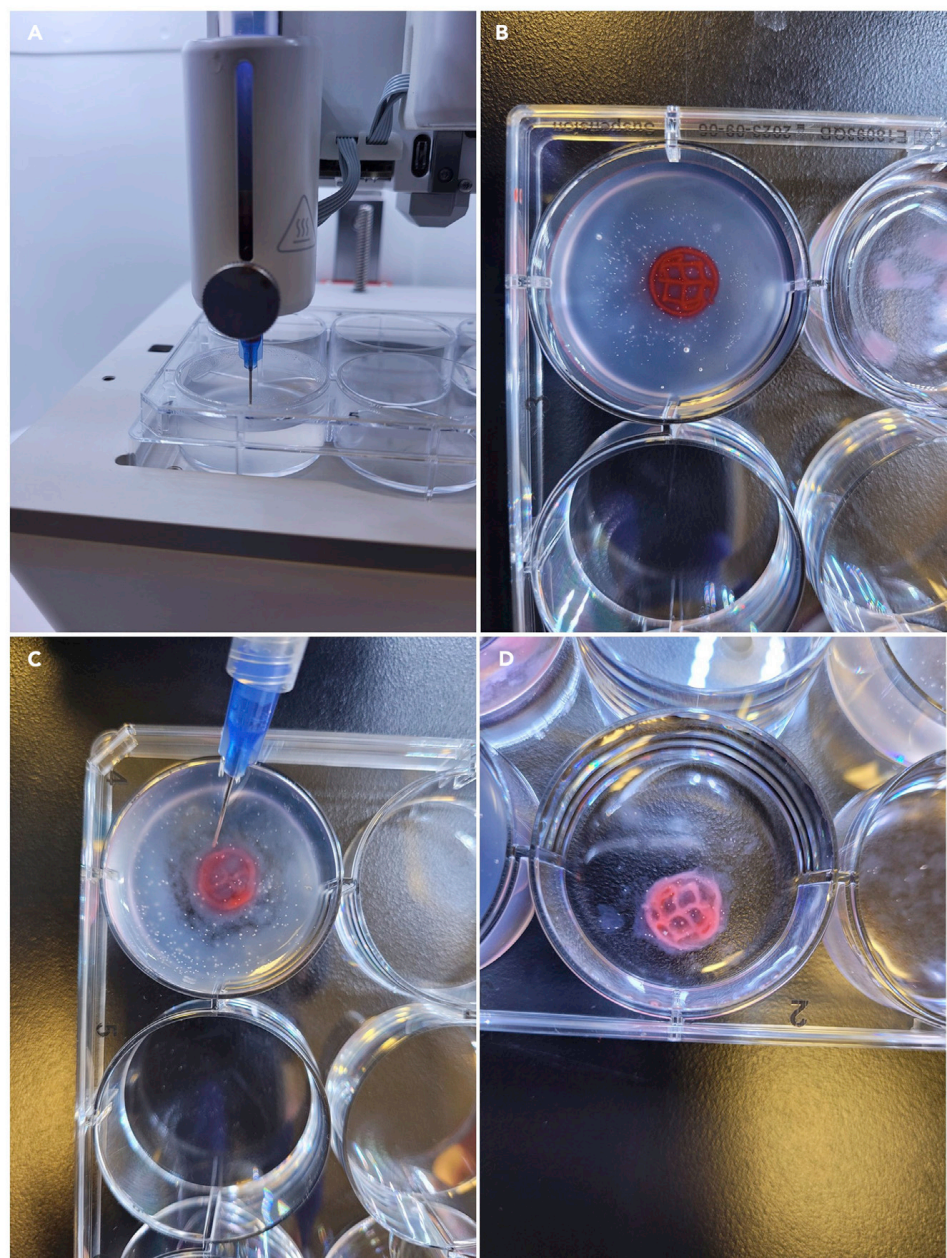
The process of printing into an agarose support bath (A) Needle tip is calibrated to sit submerged in the agarose support bath. (B) The finished bioprinted construct should have distinct fibers and hold its shape. (C) When crosslinking, the needle should circle the construct as close as possible without compromising the shape of the print. (D) The fully crosslinked construct should hold its shape outside of the support bath and the individual fibers should be clearly visible.
22. Begin the print by pressing “Print” (Figure 4B).
  a. Adjust the speed and pressure as needed to obtain a steady and even print problem 2: Lack of or inconsistent material extrusion, problem 3: Nozzle tip becomes clogged.
  b. A speed of 15 mm/s and pressure of 11 kPa is recommended.
23. Fill a syringe with crosslinker attached to a blunt tip needle.
24. Slowly inject crosslinker into the agarose gel surrounding the printed construct and into any empty spaces within the print (Figure 4C).
25. Allow it to sit for 2–3 min or until the construct has been sufficiently crosslinked. Problem 4: Printed construct does not hold its shape during printing.

***Note:*** The printed construct is fully crosslinked when the material has gelled and holds its shape (Figure 4D).

20. Fill the wells of a cell culture plate with agarose gel using a pipette.
21. Calibrate the printhead so the needle tip sits submerged in the agarose near the bottom of the plate (Figure 4A).
  a. Printing with a blunt tip needle is recommended.

26. Use a spatula to remove the print and transfer to an empty well. Problem 5: Nozzle tip leaks between prints.
27. Use a wash bottle filled with TBS to rinse excess agarose off the construct.
28. Transfer construct to desired medium for further culturing.

***Note:*** Phenol red was used in the images for visibility.

**Table 3:** Recommended printing parameters for printing into an agarose support bath

Printing parameter	Value
Calibration Height	1 mm
Print Speed	15 mm/s
Pressure	11 kPa
Crosslinking Time	2–3 min

### Printing with Alg/gel

**Timing: 30 min**

Table 4 presents the recommended printing parameters for printing with Alg/Gel or other more viscous bioinks.

29. Alg/Gel is viscous enough to be printed without a support bath or pre-crosslinking.
30. Calibrate the printhead so that the nozzle tip is approximately 0.1 mm away from the print surface.
31. Start the print by pressing “Print”.
  a. A speed of 20 mm/s and a pressure of 20 kPa is recommended.
  b. Adjust the speed and pressure as desired.
32. After printing, pipette 2% calcium chloride onto the print to crosslink.
33. Leave print in crosslinker bath for 5 min to fully crosslink.

***Note:*** Constructs are fully crosslinked when they are firm and hold their shape. Fully crosslinked prints will expand and may double in size.

34. When fully crosslinked, transfer construct to a conical or petri dish using a spatula.
35. Store construct in TBS for up to 2 weeks.

**Table 4:** Recommended printing parameters for printing with Alg/Gel

Printing parameter	Value
Calibration Height	0.1 mm
Print Speed	20 mm/s
Pressure	20 kPa
Crosslinking Time	5 min

### Culturing tissue constructs

**Timing: 30 minto1 h**

36. Thaw desired media according to manufacturer’s protocol before use.
37. Pipette up the old media using a 1 mL pipette tip.
  a. Place the pipette tip at the edge of the well furthest from the construct (if possible) to avoid taking up pieces of the construct.
  b. Pipette slowly to avoid drawing the construct into the pipette tip.

***Note:*** It is normal for constructs to degrade overtime. If performing long-term cultures of 14 days or longer, constructs may degrade to the point that they could be sucked into the pipette. Pipetting gently and as far from the construct as possible will assist with minimizing additional degradation due to pipetting.

38. Repeat for necessary wells.
39. Add desired media to well using a serological pipette.
  a. Add enough media to completely cover the construct.
  b. Add media at corner of well furthest from the construct (if possible) to avoid disrupting the construct.
40. Media changes should be performed every day, or according to culturing protocol.

## Expected outcomes

**Figure 5:**
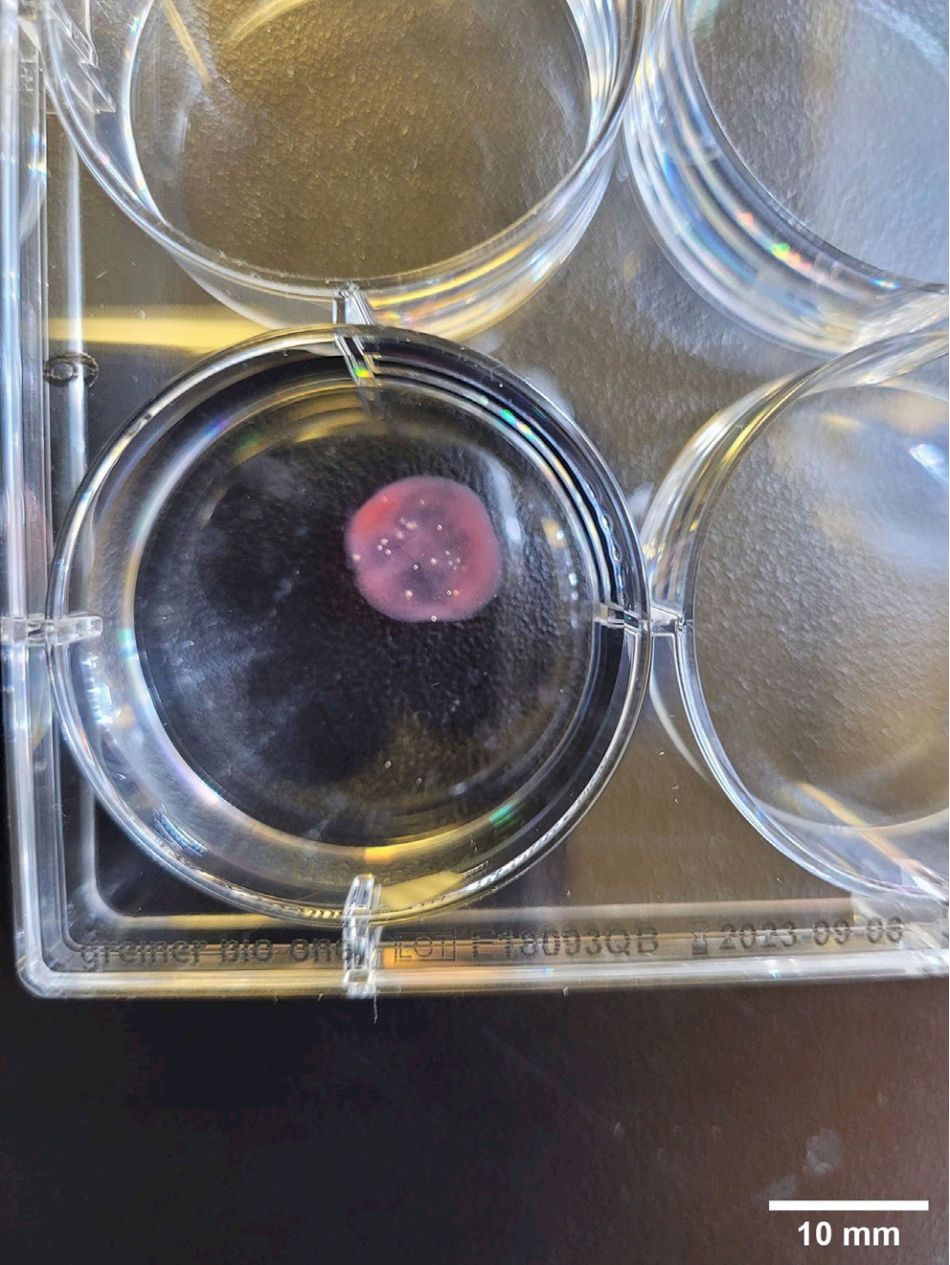
Bioprinted construct in TBS, scale bar 10 mm

The 3D printed constructs can sustain high levels of cell viability using this protocol and materials. 1 mL of bioink should produce about 3 printed constructs when printing cylindrical prints of 10 × 10 mm with 25% infill, but this is dependent on the size of construct being printed (Figure 5). Constructs are stable in a 37°C incubator for approximately 6 weeks with continued media changes. If the cell-laden constructs are fixed or the constructs are printed without cells, they can maintain stability at 4°C for approximately 6 weeks. It is expected that the printing procedures outlined in this protocol would be compatible with any extrusion-based bioink that requires crosslinking.

## Limitations

One limitation of the printing system used in this protocol is the range of material viscosities that can be used. Materials that are too viscous will clog the nozzle of the printhead, whereas significantly non-viscous materials require a support bath (such as agarose) to hold their shape during printing. Constructs printed into the agarose support bath are anticipated to hold their shape and internal structure better than constructs printed directly into a cell culture plate without agarose. It is common to see the bioink begin to spread and the fibers to merge when printing without agarose due to the time delay between printing and crosslinking the structures. Applying crosslinker after printing can result in defects in the print structure if pipetted incorrectly. Additionally, an altered pH of the crosslinker will result in the construct not fully crosslinking.

The fibrin-based bioink described here was adapted from one that has been tested with several different cell lines, including hiPSCs, NPCs, and MSCs (de la Vega et al., 2018; Abelseth et al., 2019; Sharma et al., 2020, 2021; Smits et al., 2020; Restan Perez et al., 2021; de la Vega et al., 2018; Abelseth et al., 2019; Sharma et al., 2020, 2021; Smits et al., 2020; Restan Perez et al., 2021). The ability of the bioink to maintain the viability and proliferation of other cell types may vary. Various post-processing techniques and analyses may affect the structural integrity of the constructs. Long-term culture of the 3D printed constructs may result in gradual degradation of the construct or pieces of the construct detaching. Additionally, the repeated washing steps required for fixation and staining protocols, such as in immunocytochemistry (ICC), may accelerate the degradation of the constructs.

## Troubleshooting

***Note:***Methods video S2 demonstrates the method for properly loading syringes with bioink.

Methods video S2. Demonstration of the method for properly loading syringes with bioink, related to preparing the BIO X with the bioink steps 1–3

### Problem 1

Air bubble formation.

**Figure 6:**
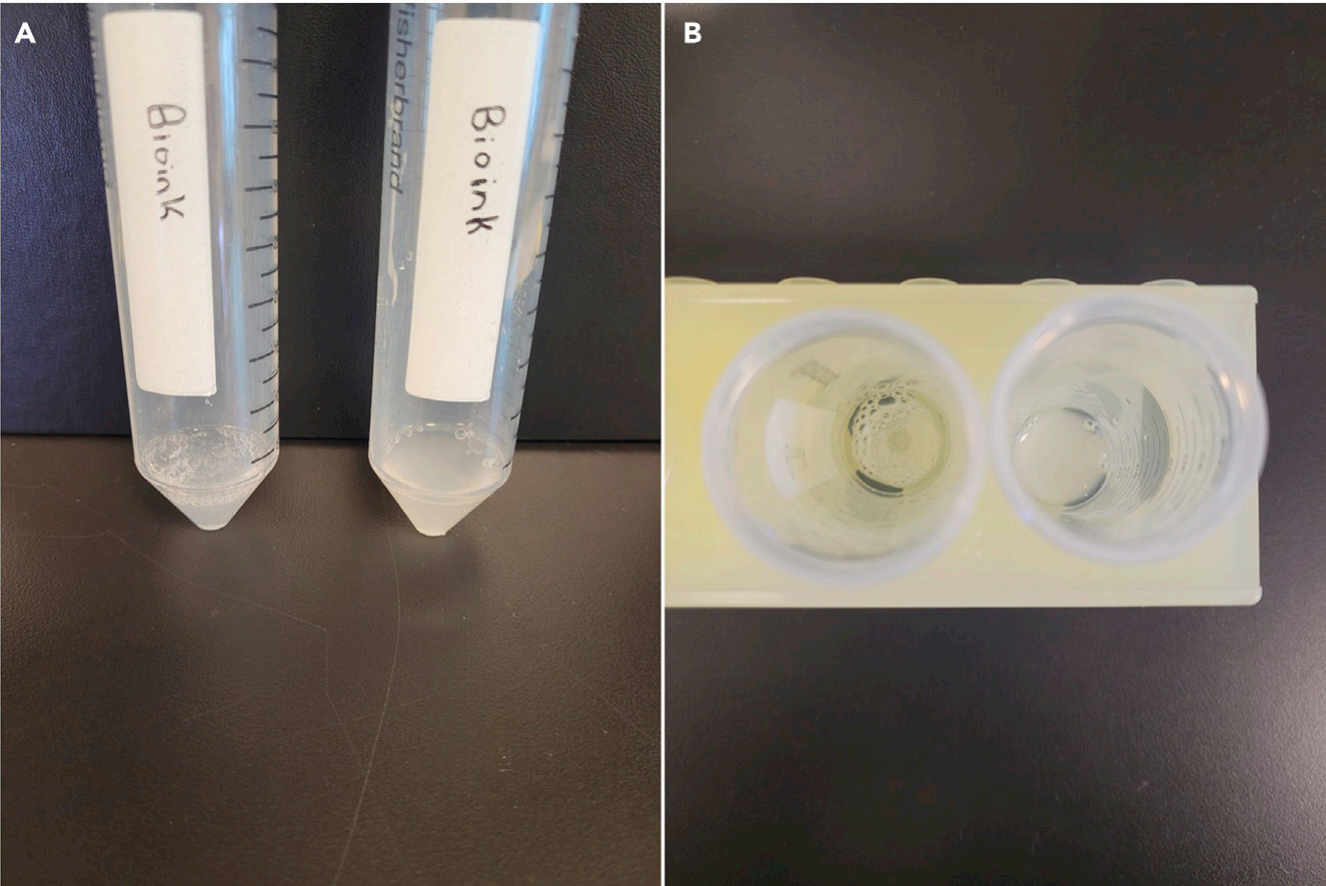
The appearance of air bubbles in bioink versus bioink without air bubbles (A) Side view of bioink with and without air bubbles. (B) Top-down view of bioink with and without air bubbles.

The formation of air bubbles while mixing the biomaterials or while loading the syringe for the printhead (encountered in preparing the BIO X with the bioink step 1c) (Figure 6).

### Potential solution

**Figure 7:**
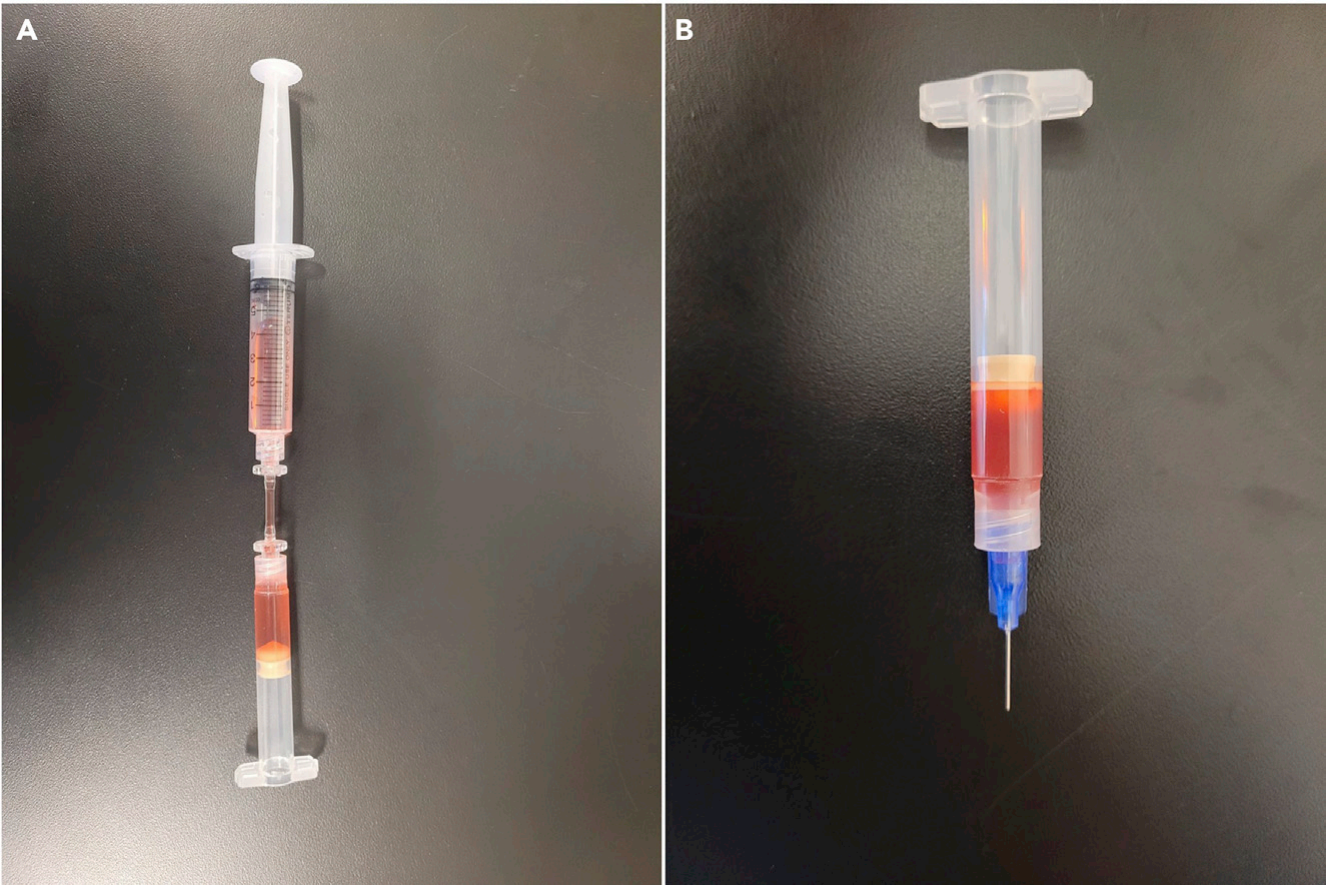
The configuration of syringes when air bubbles are present to prevent air bubble transfer (A) By placing the syringe upright, the air bubbles will be forced to the top to prevent them from being transferred between syringes. (B) Loaded syringe with no air bubbles.

• Mix slowly using a wide-bore pipette tip (cut the end off of a regular pipette tip) to avoid air bubble formation.
• Avoid expelling all the media or bioink when pipetting up and down into a solution.
• If air bubbles arise, transfer the bioink with the pneumatic syringe upside down so the bubbles stay at the surface, and avoid transferring them to the syringe while loading the printhead (Figure 7).

### Problem 2

Lack of or inconsistent material extrusion.

Issues with material extrusion during printing (encountered in printing directly onto a cell culture plate and pre-crosslinking step 15b, printing into an agarose support bath step 22a).

### Potential solution

• Increase the printing pressure by a couple of small incremental steps.
• Replace the printhead tip if it is clogged, bent, or misshapen.
• If piece 2 is not properly aligned and sealed in syringe B, then remove the bioink from the syringe and reproduce the set-up.

***Note:*** The maximum pressure that can be applied to the printhead is 200 kPa. However, pressure should be increased from the recommended starting value as little as necessary to achieve a steady flow. Excess pressure can subject the cells to shear stress and reduce the viability of the constructs.

### Problem 3

Nozzle tip becomes clogged.

Clogging issues that impact bioink extrusion during any of the printing steps.

### Potential solution

• Use a pipette tip to remove the clot from the nozzle tip.
• Slowly increase the system pressure until the clot dislodges or the bioink begins to extrude.
• Print with the needle tip further away from the build plate to prevent crosslinking with the tip.

### Problem 4

Printed construct does not hold its shape during printing.

**Figure 8:**
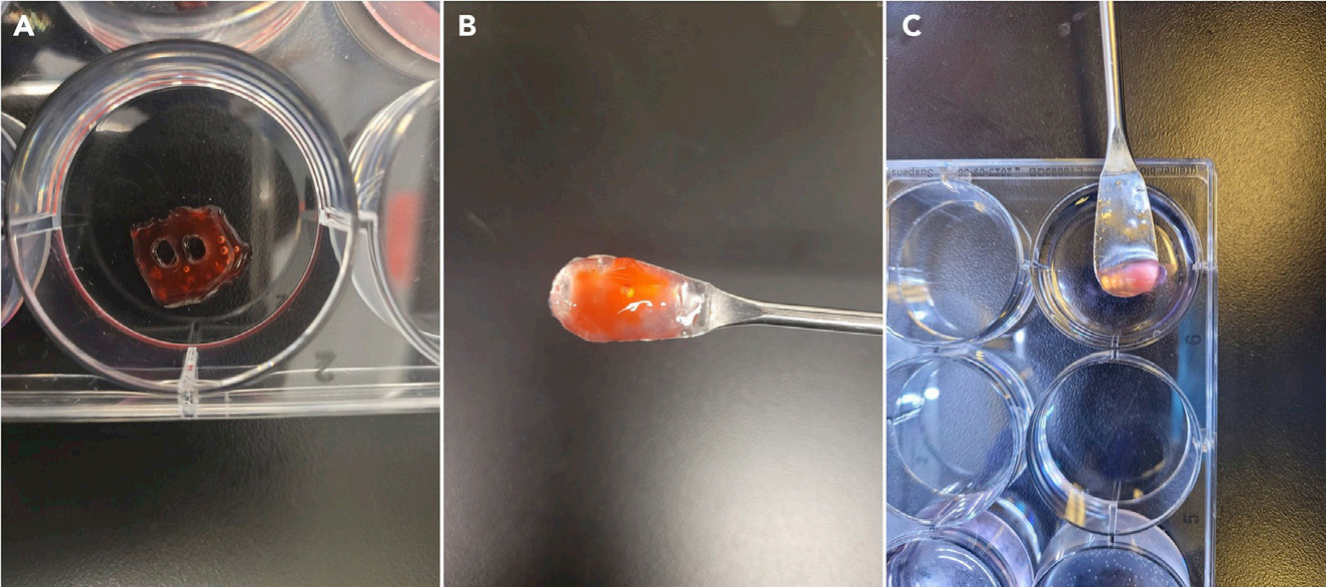
Constructs that did not hold their shape during printing (A) Fibers that have run together while printing can appear thick and uneven and cause the print to not be a uniform shape. (B) Constructs that do not hold their shape when printing into an agarose support bath will break apart upon being removed from the bath. (C) Constructs that break apart due to insufficient crosslinking could break apart into semi-crosslinked clumps.

Poor structural integrity of printed constructs when printing (encountered in printing directly onto a cell culture plate and pre-crosslinking step 15, printing into an agarose support bath step 25) (Figure 8). For comparison, Figure 5 shows a correctly printed construct.

### Potential solution

If pre-crosslinking:

• Coat the well with crosslinker before printing.
  ○ Add approximately 1 mL of crosslinker (per well in a cell culture plate).
  ○ Let sit for 2 min.
  ○ Remove crosslinker using a pipette.

If printing in an agarose support bath:

• Increase shear-thinning rate by increasing the speed.
  ○ Increase the speed incrementally until the print begins to hold its shape.
• Add more crosslinker to the construct after printing.
• Allow the crosslinked construct to sit longer before removing from support bath.

### Problem 5

Nozzle tip leaks between prints.

Any bioink leakage during printing, encountered either when printing directly into a cell culture plate or when printing into an agarose support bath.

### Potential solution

• Decrease the system pressure slightly until it ceases.
• Wipe the needle tip with a Kimwipe™ if it occurs infrequently and does not affect printing.
• Detach the pressure tube from the syringe between prints to decrease the amount of pressure pushing on the bioink.
  ○ Reattach the pressure tube just before printing.

## Resource availability

### Lead contact

Further information and requests for resources and reagents should be directed to and will be fulfilled by the lead contact, Stephanie M. Willerth (willerth@uvic.ca).

### Materials availability

This study did not generate any new unique reagents.

## Data Availability

This study did not generate or analyze datasets or code.
